# *Staphylococcus aureus* toxins mediate endothelial Thrombomodulin release during severe invasive infections

**DOI:** 10.1080/21505594.2025.2605767

**Published:** 2025-12-17

**Authors:** Lisa Seidner, Emi Tanaka, Olivia Engstrand, Sara Nilsson, Anna Bergonzini, Lara Kaland, Monica Novello, Mattias Svensson, Anna Norrby-Teglund, Laura M. Palma Medina

**Affiliations:** a Center for Infectious Medicine, Department of Medicine Huddinge, Karolinska Institutet, Karolinska University Hospital, Stockholm, Sweden; b Department of Microbiology, Faculty of Pharmacy, Meijo University, Nagoya, Japan

**Keywords:** Thrombomodulin, α-toxin, ADAM10, *Staphylococcus aureus*, necrotizing soft tissue infections

## Abstract

Thrombomodulin (TM) is a membrane protein with significant roles in coagulation hemostasis and immune response. Its soluble form (sTM) has recently emerged as a key biomarker for severe invasive bacterial infections, including Necrotizing Soft Tissue Infections (NSTI). While various mechanical, chemical, and enzymatic mechanisms have been linked to TM shedding, this study investigates the direct impact of bacterial stimuli on soft tissue cells as primary sources of TM release. We stimulated organotypic models, composed of fibroblast and endothelial cells, with NSTI clinical isolates and found that while Group A Streptococcus and *Escherichia coli* had minimal effect on TM release, *Staphylococcus aureus* infection triggered a significant increase of sTM levels. We further assessed whether the secreted proteins of *S. aureus* led to higher TM levels by increased expression, increased cell toxicity, or direct cleavage of TM from the endothelial cell membrane. To investigate these mechanisms, we performed *in vitro* stimulations of endothelial monolayers with secreted proteins of two *S. aureus* isolates differing in their *agr*-system functionality. Our results indicate that *S. aureus agr*-regulated proteins induce TM shedding by direct cleavage from the cell membrane, an effect that was inhibited by metalloproteinase inhibitors. Stimulation with the pore-forming protein α-toxin showed similar results, suggesting a potential involvement of ADAM10 in TM cleavage. Additionally, we observed that other *agr*-regulated proteins can cleave TM directly. Altogether, this study reveals a pathogen-specific mechanism for TM release during *S. aureus* invasive infection, contributing to its elevated plasma levels and providing deeper insights into the pathophysiology of NSTI.

## Introduction

Thrombomodulin (TM) is a type-I transmembrane glycoprotein, which is traditionally known for its fibrinolytic and anticoagulative role in hemostasis [1,2]. The complex formed by its binding to thrombin activates protein C and enhances the activation of thrombin-activatable fibrinolysis inhibitor (TAFI) [3]. TM has additional functions as a regulator of the immune response [4], by binding to and inactivating components of the complement system and interacting with the high-mobility group box 1 (HMGB1), reducing its pro-inflammatory effects [5,6]. TM has also been reported to bind to lipopolysaccharides (LPS), which are components of the outer membrane of Gram-negative bacteria and potent pro-inflammatory stimuli [7,8].

TM can be expressed by multiple cell types; however, it is primarily found on endothelial cells lining the blood and lymph vessels [8–10]. TM can also be found circulating in blood in its soluble form (sTM). Several different mechanisms have been described as causatives of TM shedding, including enzymatic cleavage by proteases such as neutrophil elastase, cathepsin G, and matrix metalloproteinases (MMPs) [11,12], as well as chemical [13] and physical cleavage [14]. Notably, shedding of TM has been associated with endothelial injury [8,9,15] and to be induced by cytokines such as TNF-α[12]. Previous studies have shown a correlation between circulating sTM and infection, including association with mortality in sepsis [16,17] and septic shock [15]. Therefore, it has been proposed that this protein could potentially be used as a biomarker for diagnosis and prognosis of sepsis patients [9].

More recently, we detected increased amounts of sTM in plasma of patients with Necrotizing Soft Tissue Infections (NSTI) [18]. We observed that these elevated levels were found in all patients, regardless of the etiology of infection or severity of symptoms. Notably, the elevated sTM concentrations in NSTI were significantly higher than in patients with similar clinical presentation but without necrotic infections (non-NSTI). Moreover, when compared to the sTM levels in sepsis patients in the intensive care unit (ICU), NSTI plasma samples also showed higher sTM concentrations, suggesting that sTM could serve as a specific diagnostic marker for NSTI. While the elevated sTM levels in sepsis can be attributed to the high degree of vascular impairment in sepsis-associated organ dysfunction [15], the source and mechanism for TM release in NSTI still require further investigation.

In this study, we investigated whether bacterial stimuli could directly induce TM shedding, aiming to uncover specific mechanisms underlying the elevated levels of sTM observed in NSTI, independently of immune cell involvement. NSTI clinical isolates of Group A Streptococcus (GAS), *Staphylococcus aureus*, and *Escherichia coli* were used to infect an organotypic 3D models composed of human fibroblasts and endothelial cells to assess their capacity to induce the release of TM. Our results showed that not all pathogens triggered sTM release and only *S. aureus* induced a significant increase. Since the endothelium has been described as the primary source of TM in sepsis, we focused on identifying the mechanisms by which *S. aureus* proteins stimulate sTM shedding from endothelial cells. We assessed the potential role of *S. aureus* proteins regulated by the accessory gene regulator (*agr*) system on TM shedding by using two isolates with either a functional or an impaired *agr* system. Protease inhibitors were used to explore the involvement of metalloproteinases and ADAM10, the latter of which is activated by the *agr*-regulated pore-forming α-toxin. Our results showed that metalloproteinase-mediated cleavage, triggered by *S. aureus* proteins, contributes to the observed increase in sTM during *S. aureus* infections in soft tissue.

## Materials and methods

### Bacterial culture

We used three clinical bacterial isolates from the INFECT cohort [19], all obtained from patients with confirmed NSTI: *Streptococcus pyogenes* (Group A Streptococcus, GAS; isolate 2006, sampled from blood), *Staphylococcus aureus* (isolate 2023, sampled from deep tissue) and *Escherichia coli* (isolate 4011, sampled from blood). The samples were collected for diagnostic purposes at admission or during exploratory surgery. The INFECT study is registered at ClinicalTrials.gov (NCT01790698) and was approved by national ethics committees: the Regional Ethical Review Board at the Karolinska Institute in Stockholm, Sweden; the regional Ethical Review Board at the National Committee on Health Research Ethics in Copenhagen, Denmark; the Regional Ethics Committee in Gothenburg, Sweden; and the Regional Ethics Committee in Vest, Norway. Two additional *S. aureus* strains were used: M37, isolated from recurrent skin infection and PUNE08 (P08), isolated from an acute soft tissue infection [20]; both were obtained from tissue or abscess swabs taken during hospitalization in Mumbai Hospital or Victoria Hospital (Bengaluru) in India. Ethical clearances were obtained from the respective Institutional Ethics Committees at the hospitals. All clinical isolates have been previously used by the research group in earlier projects [18,20,21] and are available at the local biobank. In addition, some experiments included *Lactococcus lactis* (commercial strain NZ9000) as a nonpathogenic control.

Bacterial strains were streaked and grown on blood agar plates overnight and thereafter one CFU was inoculated and cultured overnight (16 h) at 37 °C in 10 ml liquid media. GAS was cultured in Todd-Hewitt Broth with 1.5% yeast extract, *S. aureus* in CCY media and both *E. coli* and *L. lactis* in Tryptic Soy Broth. All species except GAS were cultured with agitation at 155 revolutions per minute (rpm).

Live bacteria for infection experiments were collected by centrifugation and washed with Phosphate-Buffered Saline (PBS) at 3488 × *g* for 10 min at room temperature (RT). Bacterial concentration was determined with a BactoBox® (SBT Instruments, Denmark) according to manufacturer’s protocol.

Bacterial supernatants for stimulation experiments were collected after centrifugation. The supernatants were filtered with 0.22 μm syringe filters. Protein concentrations were quantified using the Qubit™ Protein Assay Kit (Thermo Fisher Scientific, Q33211).

### Cell culture

Two primary cell lines were used: Human Umbilical Vein Endothelial Cells (HUVECs; Lonza C2519A; passage 4) cultured in Endothelial Growth Medium 2 with supplements (EGM-2, PromoCell, C-22011 and C-39216) and Normal Human Dermal Fibroblasts (NHDF; PromoCell C-12302; passage 9) cultured in DMEM (Cytiva, SH30022.FS) supplemented with 10% Fetal Bovine Serum (FBS; Merck, F7524), 1% HEPES (Cytiva, SH40003.01), 1% L-glutamine (Cytiva, SH30034.01), and 1% sodium pyruvate (Sigma, S8636). Cells were cultured at 37 °C in a humidified incubator with 5% CO_2_ with exchange of media every 2 d. Cells were split at least once before seeding for infection or stimulation.

The 3D organotypic soft tissue model was built in transwells (Falcon, 353046). First, the outer side of the transwells was coated with collagen at a concentration of 50 µg/ml (Advanced Biomatrix, 5005) for 2 h at RT and washed with PBS afterward. A total of 1 million HUVECs (Lonza C2519A; passage 6) were added on top of the collagen coating and incubated at 37 °C, 5% CO_2_ for 2 h in a humidity chamber. Following incubation, the transwells were placed in 6-well plates, and the HUVEC layer was cultured in EGM-2 for 72 h, changing media every 48 h. The seeding of the fibroblast layer was performed as previously described [22,23]. In brief, the model is placed in the inner side of the transwell and consists of an acellular collagen matrix (51% DMEM, 0.05 mg/mL Gentamicin, 1.75 mg/mL NaHCO_3_, 9% (v/v) FBS, 0.82% (v/v) L-glutamine, and 69% (v/v) Collagen) polymerized for 30 min at 37°C, 5% CO_2_, followed by a cellular layer (51% (v/v), 0.05 mg/mL Gentamicin, and 1.75 mg/mL NaHCO_3_, 9% FBS, 0.82% L-glutamine, 67% Collagen, and 40,000 NHDF cells) which is incubated for 2 h at 37°C, 5% CO_2_. Models were incubated in 50% EGM-2 and 50% complete DMEM for 10 d prior to infection.

For stimulation assays, HUVECs (Lonza C2519A; passage 6) were seeded at a density of 20,000 cells per well in 24-well tissue culture plates and cultured for 4 d to reach confluence before treatment with a media change after 2 d.

### *In vitro* infection

Models were infected after 14 d of culture from the day of endothelial seeding. Before infection, media was removed from all models, and 1 ml of fresh media was added to the bottom of the well. Models were infected on top of the fibroblast layer with 250,000 bacteria/model or with PBS for a mock infection as control and incubated for 22 h at 37 °C, 5% CO_2_.

After the infection or stimulation period, the cell culture supernatants of all samples were collected and centrifuged at 3488 × *g* for 10 min at RT. The cell culture supernatants were kept at −80 °C for further measurement. For microscopy of the endothelial layer, the collagen layer was removed, and the transwell membranes with the endothelial monolayer were fixed in 4% paraformaldehyde for 15 min at RT and stored in PBS at 4 °C until staining.

### Immunofluorescence microscopy

Previously fixed membranes with the endothelial monolayer were cut out with a scalpel from the transwell plastic support and permeabilized with a solution of 0,5% saponin and 20% FBS in PBS for 30 min at RT. Permeabilization buffer was removed, and samples were blocked with 2% bovine serum albumin (BSA), 0.1% saponin, 20% FBS and 1% donkey serum (Merck, D9663) in PBS. After 1 h, the buffer was removed, and samples were stained with Thrombomodulin mouse monoclonal antibody (Invitrogen, MA5-11454, diluted 1:100), Anti-*Streptococcus pyogenes* Group A Carbohydrate goat antibody (Abcam, ab9191, diluted 6,2 µg/ml), *Staphylococcus aureus* rabbit antibody (Novus Biologicals, NB100-64499, diluted 2,7 µg/ml), and *Escherichia coli* goat antibody (Bio-Rad, OBT0986, diluted 20 µg/ml), diluted in wash buffer containing 2% BSA, 0,1% saponin and 20% FBS. Samples were incubated with primary antibodies overnight at 4 °C. Samples were washed three times with wash buffer and incubated with secondary antibodies for 1 h at RT. Secondary antibodies were diluted to a final concentration of 2 µg/ml and included Donkey anti-Goat IgG (H+L) Cross-Adsorbed Secondary Antibody, Alexa Fluor™ 594 (Invitrogen, A-11058), Donkey anti-Rabbit IgG (H+L) Highly Cross-Adsorbed Secondary Antibody, Alexa Fluor™ 594 (Invitrogen, A-21207), and Donkey anti-Mouse IgG (H+L) Highly Cross-Adsorbed Secondary Antibody, Alexa Fluor™ 647 (Invitrogen, A-31571). Samples were washed and incubated with Zona Occludens-1 (ZO-1) Monoclonal Antibody (Invitrogen, ZO1-1A12), Alexa Fluor™ 488 (Invitrogen, 339,188, diluted 0,5 µg/ml), for 1 h at RT. Lastly, samples were washed and mounted with ProLong^TM^ Gold Antifade Mounting media with DNA Stain DAPI (Invitrogen, P36935). Images were acquired using a confocal microscope (Nikon, A1R HD25) with NIS-Elements imaging software and later processed in ImageJ 1.53k. Pictures were taken in Z-stacks, and the function Z-project – maximal intensity – was used to flatten the stack. Background was removed with rolling ball radius and sliding paraboloid.

### *In vitro* stimulations

Confluent HUVEC monolayers were stimulated for 22 h at 37 °C. The experimental conditions included the following treatments: (i) negative control, (ii) *S. aureus* overnight culture supernatants from strains M37 or P08 (7,000 ng/mL protein), (iii) 50 ng/mL recombinant α-toxin (IBT Bioservices, 1401–002), and (iv) co-treatment with 1300 nM broad-spectrum MMP inhibitor Marimastat (Tocris Bioscience, 2631; CAS: 154039-60–8) or 10,6 µM ADAM10 inhibitor GI254023X (Sigma-Aldrich, SML0789) in combination with *S. aureus* supernatants or recombinant α-toxin.

### Biochemical assays for quantitative analysis

Soluble factors in supernatants from the tissue model infection were measured with a customized Human Luminex® Discovery Assay (Bio-Techne) including CCL11, CCL2, CX3CL1, CXCL10, E-Selectin, GCSF, IL1β, IL10, IL2, IL6, IL8, MMP9, S100A8, S100A9, Thrombomodulin, TNFSF6, and TSLP. The assay was run according to the manufacturer’s protocol and measured with a Bio-Plex® MAGPIX™ Multiplex Reader (Bio-Rad) and Bio-Plex Manager™ MP Software (Bio-Rad). Analytes with a high fraction of out-of-rate (OOR) readings were excluded from further analysis.

Cell culture supernatants from the stimulation assays were used for the quantification of sTM and lactate dehydrogenase (LDH) release. Supernatants were centrifuged at 3000 × *g* at RT for 3 min and assayed in duplicate. The Human Thrombomodulin DuoSet ELISA (R&D Systems, DY3947) was used according to the manufacturer’s instructions to measure sTM levels. Optical density was read at 450 nm and corrected by subtracting readings at 540 nm. LDH was measured to assess cell membrane integrity using the CyQUANT™ LDH Cytotoxicity Assay (ThermoFisher, C20300) according to the manufacturer’s protocol. Absorbance was measured at 490 nm and corrected by subtracting readings at 680 nm. Data are presented as corrected optical densities (ODs) as control samples fell below the standard curve’s lower limit and proceeding with OD values allowed statistical testing. Transcripts from the thrombomodulin (THBD) gene were quantified by quantitative real-time PCR (qPCR) using TaqMan® Gene Expression Assays. Total RNA was extracted using TRIzol® reagent (Invitrogen, 15596026) and purified with RiboPure® kit (Invitrogen, AM1924) following manufacturer’s instructions. RNA concentration was determined using the Qubit® RNA HS assay kit (Invitrogen, Q32852). Reverse transcription was performed using the SuperScript™ IV VILO™ Master Mix with ezDNase™ Enzyme (Invitrogen, 11766050), which includes a DNase treatment step to eliminate contaminating genomic DNA. qPCR reactions were prepared using TaqMan® Fast Advanced Master Mix (Applied Biosystems, 4444556) and TaqMan® Gene Expression Assay specific for THBD (Life technologies; Hs00264920_s1 FAM) and the endogenous control gene 18s (Life technologies; Hs99999901_s1 VIC_PL). Amplification was carried out on a QuantStudio5 (Applied Biosystems) under the following cycling conditions: initial activation at 50 °C for 2 min and 95 °C for 20 s, followed by 40 cycles of denaturation at 95 °C for 1 s and annealing/extension at 60 °C for 20 s. Each sample was run in technical duplicates. Relative gene expression was calculated using the comparative Ct (ΔΔCt) method, with normalization to 18 s.

### TM degradation assay

Forty nanogram of recombinant human TM (rTM; Prepotech, 100–58) was mixed with 100 ng of recombinant human ADAM10 (rADAM10; R&D biotechne, 936-AD) and assay buffer containing 50 mM Tris, 150 mM NaCl, 10 mM CaCl2 and 1 mM ZnCl2, pH 7.5, and incubated for 2 h at 37 °C. Samples containing metalloprotease inhibitors Marimastat at 1300 nM and ADAM10 inhibitor GI254023X at 10.6 µM were pre-incubated with recombinant ADAM10 and assay buffer for 30 min at RT before rTM was added. rTM 50 ng was mixed with undiluted *S. aureus* overnight culture supernatant and incubated for 22 h at 37 °C. Protease inhibitors (G-Biosciences, 786–207), 4-(2-Aminoethyl)-benzenesulfonylfluoride hydrochloride (AEBSF), Aprotinin, Calpain Inhibitor I (ALLN), L-trans-Epoxysuccinyl-leucylamide-(4-guanido)-butane (E-64) and EDTA-Na2, were incubated for 15 min at 37 °C with bacterial supernatant individually at their highest recommended working concentration prior to incubation with rTM.

The samples for SDS-PAGE were prepared with NuPAGE™ LDS sample buffer (Invitrogen, NP0008) and NuPAGE™ Sample Reducing Agent (Invitrogen, NP0004) and heated at 70 °C for 10 min. Samples and PageRuler Plus Prestained Protein Ladder (Thermo Fisher Scientific, 26,619) were loaded on 4–12% NuPAGE™ Bis-Tris gels (Invitrogen, NP0321BOX). Separation was performed by electrophoresis using NuPAGE MES SDS running buffer (Invitrogen, NP0002) containing NuPAGE antioxidant (Invitrogen, NP0005) at initially 50 V for 15 min, followed by 200 V for the remainder of the run. Gels were blotted to nitrocellulose membranes (Invitrogen, IB23002) using the iBlot 2 dry blotting system (Invitrogen, IB21001). After transfer, membranes were washed in deionized water for 5 min at RT and blocked in 5% skim milk in TBS with 0.05% Tween (TBS-T) for 1 h followed by incubation with 1:1000 anti-human thrombomodulin primary antibody (ProteinTech, 14,318–1-AP) in 5% milk TBS-T for 1 h at RT. Membranes were washed in TBS-T and incubated with 1:2500 secondary HRP-coupled antibody for 30 min at 37 °C. Membranes were washed in TBS-T and incubated with substrate (Thermo Scientific, 34579). Detection of bands was carried out with Odyssey Fc Imager (LI-COR). The images were processed with ImageStudio Software (v. 6.1.0.79) and merged with ImageJ (v. 1.54p).

### Statistical analyses

Statistical analyses were performed in GraphPad Prism (v. 10.0.2). Analysis of soluble factors was performed by Analysis of Variance (ANOVA) with matched samples. Fisher’s LDS test was used for multiple comparisons on the entire datasets. For pairwise comparisons of LDH or sTM measurements, a paired two-tailed Student’s *t*-test was performed.

## Results

### *Staphylococcus aureus* infection stimulates the release of sTM in vitro

In our previous study [18], we identified sTM in plasma as a potental biomarker for identification of NSTI. Elevated concentrations of sTM were observed in all NSTI cases, regardless of etiology, compared to patients suspected of NSTI but without necrotic tissue evidence upon surgical exploration (non-NSTI) (Supplementary figure S1). Consequently, in this study, we aimed to investigate the mechanisms underlying sTM release in response to direct bacterial stimulation.

**Figure 1.**
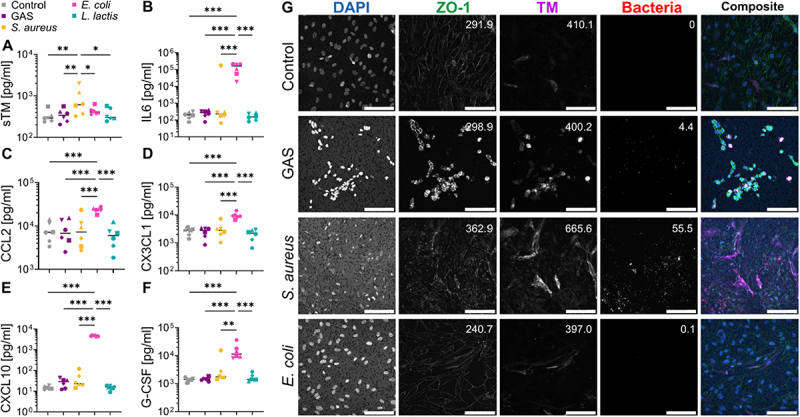
Response of 3D tissue models infected with NSTI clinical isolates and one nonpathogenic bacterial strain. The tissue models composed of human fibroblasts and endothelial cells were stimulated for 22 h with live bacterial cultures. The effect of the infection was assessed by measurement of sTM (A), IL-6 (B), CCL2 (C), CX3CL1 (D), CXCL10 (E) and G-CSF (F) in media supernatant by Luminex multiplex assay. Different replicates are shown with distinctive symbols (*n* = 6). The differences were evaluated with a repeated measurements ANOVA followed by Fisher’s least significant difference (LSD) post hoc test. Stars represent significant differences: *p-value < 0.05, **p-value < 0.01 and ***p-value < 0.005. The presence and effect of the bacteria in the endothelial layer was observed through immunofluorescence confocal microscopy (G). We assessed the integrity of the endothelium with zona-occludes 1 (ZO-1, green), the protein expression of TM (purple) and bacterial presence (red). Mean fluorescence intensity values for each channel are shown in the upper right corner of each micrograph. The control condition involved a mock infection with PBS. Scale bar = 100 µm.

We used a 3D tissue model that includes fibrobasts and HUVECs to identify the potential source of sTM in soft tissue infections. This model is similar to a previously developed one that has proven successful in the study of NSTI [22,23]. We performed 22 h infections with NSTI isolates of GAS, *S. aureus* and *E. coli*, which are frequent NSTI causative species. Their effects were evaluated by the quantification of selected proteins in the cell culture media supernatants after infection. We observed that *S. aureus* was the only isolate that elicited increased concentrations of sTM in the supernant (Figure 1(A)). However, the inflammatory markers IL-6, CCL2, and CX_3_CL1 did not show a significant increase following *S. aureus* infection. Instead, a strong inflammatory response was only triggered by *E. coli* infection (Figure 1(B–D)). In our previous work [18], we identified six additional proteins for identification of mono-/poly-microbial etiology and septic shock. In the current study, we evaluated these six markers in cell culture media after stimulation of the soft tissue model. Of these, only CXCL10 and G-CSF were measurable, showing high levels only after infection with *E. coli* (Figure 1(E-F)).

Since endothelial cells are one of the main producers of TM [8], we evaluated the migration of bacteria to the endothelial layer and its TM expression using confocal immunofluorescence microscopy (Figure 1(G)). We observed that GAS was present at the endothelial layer, completely disrupting the monolayer by inducing death of the host cells. In comparison, *S. aureus* was detected in greater numbers than GAS at the endothelial layer, but despite this higher baterial load, the endothelial cells were still present. Nonetheless, in this infection the cells’ nuclei displayed altered morphology, and the integrity of the monolayer was compromised, as evidenced by the disrupted ZO-1 localization at the cell membranes. In agreement with our quantification in host cell culture supernatants post-infection, *S. aureus* infection showed the highest TM expression as compared to the other bacteria. Lastly, we did not observe major disruption of the endothelial layer or greater amounts of TM in the infection with *E. coli*, despite media concentrations indicating a strong inflammatory response from the cells.

Based on the higher TM protein expression observed in the endothelial layer of the tissue model, we opted to assess sTM release after stimulation of endothelial cells alone. Since most virulence factors from the selected bacterial pathogens are secreted, we stimulated a monolayer of endothelial cells with bacterial supernatant from overnight cultures. Consistent with our previous observations, *S. aureus* stimulations were again the only condition that resulted in higher concentrations of sTM (Supplementary Figure S2).

### Endothelial-derived sTM after stimulation with *S. aureus* proteins is mediated by metalloprotease dependent cleavage mechanisms

**Figure 2.**
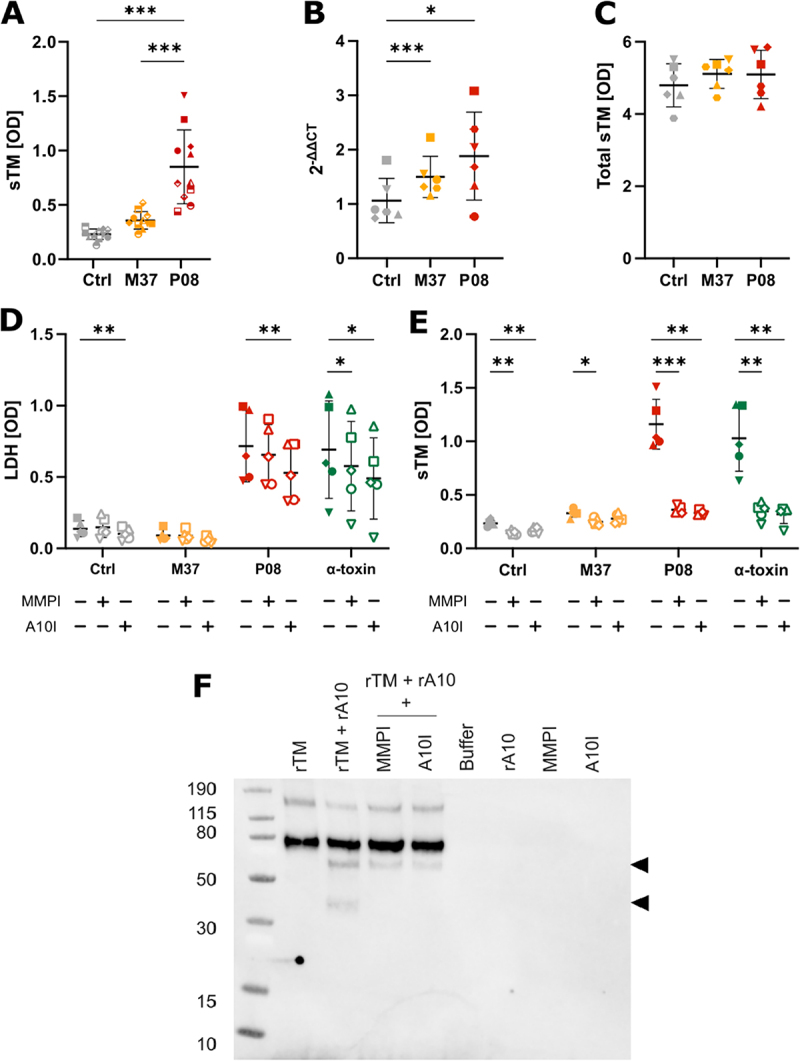
Assessments of mechanisms leading to increased sTM levels following stimulation with *S. aureus* proteins. HUVEC monolayers were stimulated for 22 h with supernatant of overnight cultures of clinically isolated strains of *S. aureus* (M37, P08) and α-toxin. sTM was assessed in the host cell culture supernatant of stimulated cells (A). Thrombomodulin gene (THBD) expression in HUVECs after stimulation was assessed by qPCR (B) and total TM levels were measured after cell lysis of stimulated cells (C). LDH and TM were measured in cell culture supernatants after stimulations, with and without addition of 1300 nM broad-spectrum MMP inhibitor Marimastat (MMPI) or 10,6 µM ADAM10 selective inhibitor GI254023X (A10I) (D-E). LDH release measurements indicate cytotoxicity. Quantification of sTM and LDH are given in corrected optical density values. Asterisks indicate statistically significant differences (* = *p* < 0.05, ** = *p* < 0.01, *** = *p* < 0.001) according to ANOVA followed by Fisher’s least significant difference (LSD) post hoc test (A, B, C) or student t-test (D, E). Colors indicate different stimuli and symbols indicate different biological replicates. Direct cleavage of 40 ng rTM by 100 ng rADAM10 (rA10) was evaluated by western blot after 2 h incubation at 37 °C, with or without MMPI or A10I (F). Cleavage products are indicated by arrows at approximately 60 and 40 kDa.

We decided to further investigate the factors that promote the elevated sTM after stimulation with *S. aureus*. To assess the potential impact of known virulence factors produced by *S. aureus*, we selected two strains isolated from skin and soft tissue infections, P08 and M37. These isolates have the same sequence type (ST) ST22 but differ in their accessory gene regulator (*agr*) quorum-sensing system due to a point mutation in the *agrC* gene [21]. As a result of this mutation, M37 has a low production of virulence factors and, therefore, is less cytotoxic than P08. We stimulated monolayers of HUVECs with bacterial overnight culture supernatants from the two selected *S. aureus* strains or left untreated for 22 h. After incubation, the levels of sTM were measured in host cell culture media. We observed that the supernatant of both M37 and P08 induced an increase in sTM levels; however, only P08 elicited a significantly higher sTM release (Figure 2(A)).

To test if the stimulation of HUVECs with *S. aureus* overnight culture supernatant affected the gene expression of TM, we quantified the *THBD* mRNA transcripts after stimulation. The results showed an upregulation of TM gene expression in response to *S. aureus* stimulation compared to the untreated control, but there were no significant differences between the strains (Figure 2(B)). Nonetheless, this observed upregulation in gene expression was not reflected on increased levels of TM protein levels. Quantification of total protein concentrations after cell lysis (Figure 2(C)) showed no significant differences in levels of TM between stimulations and control or between strains.

Since TM production remains unchanged after stimulation, we investigated whether TM shedding is a result of increased cellular death or heightened proteinases activity. To address this, we measured LDH release to assess cytotoxicity alongside the TM quantification. Among the virulence factors of *S. aureus* regulated by the *agr* system, the pore-forming α-toxin stands out for its cytotoxic capacity. Therefore, we evaluated its contribution by also including stimulation with recombinant α-toxin. Stimulations were performed with the addition of a broad spectrum MMP inhibitor (MMPI) Marimastat and the selective ADAM10 inhibitor (A10I) [24] GI254023X, as ADAM10 serves as the host receptor for α-toxin [25].

The LDH quantification showed that stimulation with the bacterial culture supernatant of M37 did not induce a cytotoxic effect, whereas stimulation with bacterial culture supernatant of P08 led to higher LDH concentrations, in agreement with its cytotoxic phenotype (Figure 2(D)). This effect was reduced by treatment with both inhibitors; however, only A10I decreased LDH concentration significantly. We also observed a cytotoxic effect in treatments with recombinant α-toxin, which was partially, yet significantly, reduced with both inhibitors MMPI and A10I.

MMPs and α-toxin-mediated cleavage of TM was assessed by inhibition of proteases. In unstimulated cells, we observed an inhibitory effect on sTM release from both MMPI and A10I, affecting the baseline release of TM in untreated conditions. These inhibitory effects were more notable in all other conditions (Figure 2(E)). M37 stimulation elicited the lowest sTM release after stimulation, but still significantly reduced by MMPI. The robust sTM release induced by P08 was also observed after stimulation with recombinant α-toxin. In both stimulations, addition of MMPI and A10I reduced the levels of sTM from endothelial cells. Our results showed that the addition of inhibitors only partially lowered the cytotoxic effect of P08 and α-toxin, while the concentration of sTM was reduced to baseline levels, suggesting that active cleavage of TM from the cell membrane is necessary for the increase of sTM levels.

Given that α-toxin stimulation alone was sufficient to elevate sTM levels, and that selective ADAM10 inhibition effectively suppressed this response, we sought to determine whether ADAM10 directly cleaves TM. To address this, we performed *in vitro* cleavage assays using recombinant TM (rTM) and recombinant ADAM10 (rADAM10), and analyzed the resulting fragments by Western blot (Figure 2(F), Supplementary Figure S3). We observed two distinct cleavage products at ~ 60 kDa and ~40 kDa, corresponding to proteolytic removal of the C-type lectin-like domain (CTLD) and cleavage at the epidermal growth factor (EGF)-like domain, respectively [26].

Taken together, these findings suggest that endothelial-derived sTM release in response to stimulation with *S. aureus* proteins involves metalloprotease-dependent mechanisms, with ADAM10 potentially contributing directly to TM shedding.

### *S. aureus* proteins can directly cleave Thrombomodulin

Given that complete inhibition of TM cleavage was not observed after addition of MMP and ADAM10 inhibitors during supernatant stimulations, we hypothesized that *S. aureus* proteases may directly cleave TM. *S. aureus* expresses several proteases, including the cysteine proteases ScpA and SspB, the serine proteases SspA and SplA-F, and the metalloprotease aureolysin (Aur), which are all secreted during infection. To investigate their potential to cleave TM directly, we incubated rTM with the supernatants of *S. aureus* overnight cultures for 22 h in the presence or absence of 5 different protease inhibitors: AEBSF and aprotinin, serine protease inhibitors; ALLN and E-64, cysteine protease inhibitors; and EDTA-Na2, metalloprotease inhibitor. TM cleavage was evaluated by western blot.

**Figure 3.**
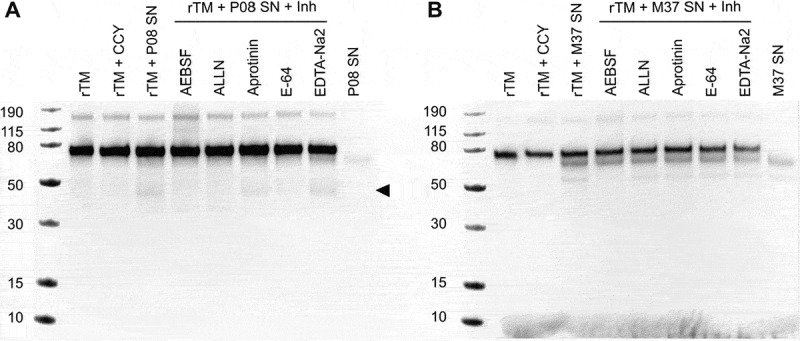
Evaluation of direct TM cleavage potential of *S. aureus* secreted proteins. Recombinant TM (rTM) was incubated for 22 h at 37 °C with overnight bacterial supernatant (SN) of P08 (A) or M37 (B) grown in CCY medium. TM cleavage products were assessed by western blot. Cleavage by different types of proteases was inhibited by addition of five different inhibitory compounds (Inh): AEBSF and aprotinin, serine protease inhibitors; ALLN and E-64, cysteine protease inhibitors; and EDTA-Na2, metalloprotease inhibitor. The arrow indicates the new cleavage product in response to bacterial supernatant, which was absent in both the rTM-only and the bacterial supernatant-only controls.

Incubation with P08 supernant generated a band of around 40 kDa (Figure 3(A)). Interestingly, incubation with both cysteine protease inhibitors lead to disappearance of the cleavage product, while the same effect was only observed in stimulation with one of the serine inhibitors. In contrast, we did not observe the same cleavage product after incubation with M37. Instead, we observed unspecific binding of the antibody to proteins in the supernatant of M37 (Figure 3(B)). These findings suggest that *S. aureus* proteases can directly cleave TM and may thereby contribute to the TM shedding observed during bacterial infection, independently of host-derived proteolytic mechanisms.

## Discussion

In this study, we investigated the direct effects of bacterial isolates from NSTI on the release of TM during infection in a tissue model with human fibroblasts and endothelial cells. Our results revealed that, among the prevalent causative bacterial agents of NSTI, *S. aureus* was the only isolate that directly affected endothelial cells, leading to the shedding of TM. This release was a direct consequence of *S. aureus* virulence factors regulated by the *agr*-regulatory system, with α-toxin being a key contributor. The findings indicate that the elevated release of TM is not due to increased protein expression or heightened cell death, but rather a result of direct cleavage of TM. Additionally, we observed that secreted proteases from *S. aureus* have the potential to directly cleave TM, further elucidating the mechanisms of TM release during severe bacterial infection.

While *S. aureus* was the only isolate that led to an evident release of TM, this infection did not elicit a heightened inflammatory response from the endothelium. Instead, *E. coli* infection triggered a robust response in the endothelial cells, as evident by elevated levels of IL-6, CCL2, and CX_3_CL1. This heightened response is likely a consequence of toll-like receptor 4 (TLR-4) signaling after binding of lipopolysaccharides (LPS) [27–31]. These cytokines and chemokines are key for the recruitment of immune cells to the site of infection and have been observed in elevated concentrations in bacterial infections [18,32,33]. Notably, the detection of these responses following infection with *E. coli* suggests that endothelial cells likely play a significant role in infections caused by this pathogen. However, it remains unclear why other etiologies did not trigger similar responses. In GAS infections, the endothelium was observed to be severely affected, with widespread cell death observed by microscopy, which would have been typically associated with high levels of proinflammatory cytokines [34]. However, we did not observe this in our results. It is possible that the source of these cytokines originates from different cell types, highlighting the complexity of the inflammatory responses in soft tissue infections.

Interestingly, it has been reported that LPS stimuli can lead to cleavage of TM on monocytes [35]. However, our observations did not show that stimulation with *E. coli* elicited such a response in endothelial cells, indicating that the mechanisms required for TM cleavage might vary depending on the cell type. Conversely, cytokine stimulation with IL-1β and TNF-α has been reported to downregulate TM expression [36–38]. Since our results show an elevated inflammatory response from *E. coli* infection, it is possible that the lack of sTM in this condition was due to lower expression levels. This indicates that the expression and shedding of TM are complex, potentially involving cell type-specific mechanisms and varying responses to different stimuli.

In order to elucidate the mechanism underlying the elevated sTM observed during *S. aureus* infection, we focused on the effects of bacterial secreted proteins on the endothelium. We investigated whether this elevation was due to increased TM expression, increased cell death, or active cleavage. Our experiments demonstrated that the elevated sTM was not attributable to higher amounts of total TM protein produced, neither due to increased cell death. Instead, it was linked to cleavage by MMPs as shown by the decreased sTM levels in media after inhibition by MMP and ADAM10 inhibitors. The effect of these inhibitors was observed in stimulations with both strains, the virulence-attenuated strain M37, and the cytotoxic strain P08.

Due to the differences in cytotoxicity from the *S. aureus* strains, we initially considered that the bacterial proteins might have a direct effect on the integrity of the cell membrane leading to the release of TM. While this effect could have been caused by multiple virulence factors regulated by *agr*, we evaluated α-toxin, a pore-forming protein that plays a key role in *S. aureus* pathogenesis. This protein has a direct cytotoxic effect [39] and has been shown to modulate immune cell function in severe pneumonia [40]. We observed that stimulation with only α-toxin resulted in a significant release of LDH and sTM. However, the inclusion of MMP and ADAM10 inhibitors effectively repressed TM release, while the cytotoxic effect of α-toxin was only partly inhibited. In accordance, our experiments with GAS in the soft tissue model also showed a complete disruption of the endothelial membrane, but the levels of TM in media remained at basal levels. These results confirm that the disruption of the cell membrane does not lead to increased amounts of TM release, and instead that active cleavage is necessary for shedding of TM from the cell membrane.

Cleavage of TM is recognized as a key physiological process during wound healing and immune response regulation, and several direct protease cleavage mechanisms have been described. During wound healing, the cleavage of TM in keratinocytes by rhomboid proteases has been highlighted as beneficial, activating cell proliferation and migration [26,41]. Moreover, proteases released by activated neutrophils, such as elastase and cathepsin G, have also shown to reduce membrane-bound TM in the endothelium [12,42]. Nonetheless, among all, MMP-mediated cleavage stands out as one of the predominant processes for TM cleavage, as it is triggered by inflammatory conditions [9]. In alveolar epithelial cells, exposure to proinflammatory cytokines (TNF-α, IL-1β, and IFN-γ) led to TM shedding, which was inhibited by the MMP inhibitors TAPI and GM6001 [43]. Similar findings were observed in endothelial cells, including EA.hy926 and HUVECs, where stimulation with venom also induced TM cleavage that was blocked by the same inhibitors [11]. Additional evidence comes from experiments showing that lysophosphatidic acid induces shedding of the TM lectin-like domain in a MMP-dependent mechanism [44]. In our study, the shedding of TM induced by *S. aureus* culture supernatant was inhibited by Marimastat, a broad spectrum MMP inhibitor with confirmed activity against MMP-1, −2, −3, −7, and −9 [45], suggesting that *S. aureus* proteins induce cleavage of TM via host MMP activation. Notably, a similar inhibitory effect was observed with GI254023X, a selective ADAM10 inhibitor [24]. Therefore, it is possible that *S. aureus* stimulation may lead to a parallel or sequential activation of both ADAM10 and MMPs, resulting in potential co-occurring mechanisms contributing to TM shedding. Future studies could expand on these mechanisms by profiling the regulation and activation of these proteases during infection. Interestingly, both inhibitors also effectively blocked TM release after stimulation with α-toxin alone. This virulence factor binds to ADAM10, enabling pore formation and activating this protease, known to cleave membrane proteins such as E-cadherin [25]. These results suggest that the release of sTM from endothelial cells in response to *S. aureus* stimulation could be mediated through α-toxin-induced activation of ADAM10 protease activity. Moreover, we observed that rADAM10 cleaved rTM in a dose-dependent manner and that this cleavage was effectively inhibited by both Marimastat and GI254023X. These findings provide evidence that ADAM10 can cleave TM and further support its involvement in TM shedding during *S. aureus* infection but not excluding a possible MMP involvement occurring in parallel or downstream. To our knowledge, no prior studies have reported that ADAM10 mediates the cleavage of TM from endothelial cells, highlighting yet a potential additional effect of α-toxin in *S. aureus* infections. While our results suggest a potentially relevant role of ADAM10, further studies are warranted to confirm the functional significance of this mechanism. Genetic approaches, such as knockouts or knockdowns of ADAM10 and/or MMPs, would help delineate their individual contributions to TM shedding and reveal whether they act in a coordinated or independent manner.

The role of ADAM10 in the development of severe *S. aureus* infection has been highlighted in studies where the conditional disruption of ADAM10 or its inhibition with GI254023X have shown development of milder local infections in lung [46] and skin [47] of mouse models. These outcomes have been linked to the cleavage of E-cadherin and loss of tissue integrity. In addition to this, ADAM10’s role in coagulopathies has also been shown recently, as α-toxin stimulation was shown to induce platelet aggregation, while ADAM10 deletion limited both platelet aggregation and the resulting microvascular occlusion [48,49]. Nonetheless, the whole pathway linking α-toxin with platelet aggregation has not been described. The observed cleavage of TM induced by α-toxin-ADAM10 activation, as observed in our study, could contribute to the reduction of anticoagulatory effect of TM and potentially lead to the procoagulant condition seen during invasive infection of *S. aureus*.

The activation of ADAM10 by pore-forming protein activity is not unique to α-toxin, and it has been seen in other pathogens. Interestingly, ADAM10 deletion in a sepsis mouse model with *Pseudomonas aeruginosa* and *Streptococcus pneumoniae* showed a reduction in mortality; an effect that was not seen with Group B *Streptococcus* or *Candida albicans* [48]. The pathogen-specificity of ADAM10 activation supports our observations where the cleavage of TM was not detected in GAS or *E. coli* isolates from necrotic infections. Based on our results, the underlying mechanism and source of sTM in GAS or *E. coli* NSTI remain to be elucidated. Moreover, NSTI is caused by a wide variety of microorganisms including anaerobes [19] which were not evaluated in our study. Therefore, further experiments including additional pathogens, mixed bacterial cultures, and/or anaerobic conditions are warranted to better reflect the clinical complexity of NSTI and assess pathogen-specific TM shedding mechanisms.

TM has several domains where potential cleavage can occur, leading to at least seven different soluble fragments [9]. These different cleavage sites could determine the downstream effect of sTM and its implications on the host-pathogen interactions during infection [8]. Our results show ADAM10-mediated cleavage of TM in two regions, as evident by the appearance of cleavage products at 60 kDa and 40 kDa observed by western blot. These fragments correspond to the removal of the C-type lectin-like domain (CTLD) and cleavage within the epidermal growth factor (EGF)-like domain, respectively.

Moreover, our results show an additional TM shedding mechanism but only in endothelial cells. Thus, it is unknown if *S. aureus* infection could lead to the same effect in other TM expressing cell types, affecting the infection in different tissues. Further investigation is needed to elucidate potential interactions between different mechanisms leading to TM shedding, including the known proteolytic cleavage mediated by proteases from immune cells [11,12]. Lastly, we observed an additional TM cleavage effect directly from *S. aureus* proteins. Secreted proteins from the cytotoxic isolate P08 showed TM cleaving capacity, which were inhibited by serine- and cysteine-protease inhibitors. Since cleavage was not evident by secreted proteins of M37, it is likely that the *agr*-regulated proteins ScpA, SspB, SspA and SplA-F are involved in this mechanism. However, it is unknown how much this cleavage mechanism contributes to the total sTM quantified, neither its potential effects during infection. Therefore, future studies using bacterial mutants lacking α-toxin and/or other virulence factors, along with complemented strains, would be valuable to further clarify the role of specific bacterial proteins in TM shedding.

In this study, we have demonstrated that bacterial proteins from *S. aureus* can induce the release of TM from the endothelial cell membrane. Our findings suggest that this mechanism is potentially mediated by ADAM10, which is activated following α-toxin binding. This new understanding adds to the multiple pathogenic mechanisms used by *S. aureus*, with potential implications for both immune modulation and coagulation in the context of severe infections of soft tissues. Moreover, it provides evidence supporting the inhibition of ADAM10 as a potential therapeutic target for preserving endothelial function during invasive infection.

## Supplementary Material

Seidner_etal_TM_revision_supFigures.docx

## Data Availability

The authors confirm that the data supporting the findings of this study are available within the article and in Zenodo at https://doi.org/10.5281/zenodo.16277160 [50].
