# Supplementary material for: Staphylococcus aureus toxins mediate endothelial Thrombomodulin release during severe invasive infections
Source: Virulence. 2025 Dec 17;17(1):2605767. doi: 10.1080/21505594.2025.2605767 (PMC12721087; doi:10.1080/21505594.2025.2605767)
Supplement: Seidner_etal_TM_revision_supFigures.docx [file KVIR_A_2605767_SM2936.docx]

**
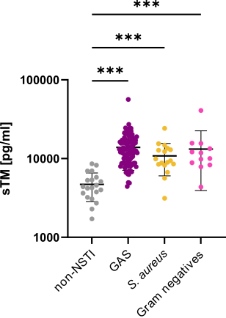
**

**Supplementary figure 1. sTM concentrations in plasma of NSTI patients and non-NSTI controls.** Data were obtained from Palma Medina et.al. (JCI, 2021) and samples were categorized based on aetiology. The levels of plasma sTM were measured in blood taken at arrival to the specialized hospital using a Luminex multiplex assay. The levels in samples from patients with NSTI were compared to those from patients with suspected NSTI but where no necrosis was found during surgical exploration (non-NSTI controls). Asterisks indicate statistically significant differences (* = p < 0.05, ** = p < 0.01, *** = p < 0.001) evaluated by Kruskal-Wallis test followed by Dunn’s post hoc test. GAS, Group A Streptococcus.


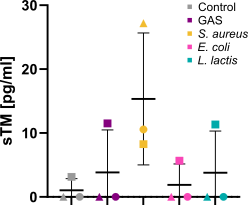


**Supplementary figure 2. sTM concentration in cell culture media of HUVECs monolayer stimulated with bacterial overnight culture supernatant or PBS.** The levels of sTM were measured by ELISA in cell culture media after 22 h stimulation. Colours indicate different stimuli and symbols indicate different biological replicates. No statistical analysis was performed as only three replicates are included.


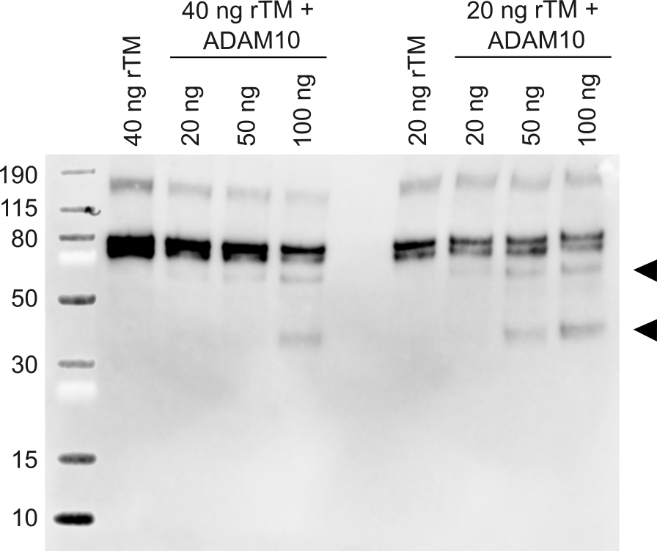


**Supplementary figure 3. Direct cleavage of rTM by rADAM10.** 40 ng or 20 ng rTM were incubated for 2 h at 37°C with three different amounts of rADAM10 (rA10): 20, 50 and 100 ng. Two cleavage products were observed by western blot, showing a dose-dependent pattern with two distinct bands at approximately 60 and 40 kDa (indicated by arrows). These bands are consistent with cleavage at the C-type lectin-like domain and the EGF-like domain, respectively.
